# High-frequency fecal indicator bacteria (FIB) observations to assess water quality drivers at an enclosed beach

**DOI:** 10.1371/journal.pone.0286029

**Published:** 2023-06-02

**Authors:** Ryan T. Searcy, Jacob R. Phaneuf, Alexandria B. Boehm

**Affiliations:** Department of Civil & Environmental Engineering, Stanford University, Stanford, California, United States of America; University of Minnesota Twin Cities, UNITED STATES

## Abstract

Fecal indicator bacteria (FIB) are monitored at beaches to assess water quality and associated health risk from recreational exposure. However, monitoring is generally conducted infrequently (i.e. weekly or less often), potentially leading to inaccurate assessment of water quality at a beach at the time of use. While some work has shown that FIB in marine environments can vary over short (e.g. subhourly) time scales, that work has been mainly focused on ‘open’ beaches. ‘Enclosed’ beaches—those that are partially barriered from exchange with offshore water and thus have different residence times and mixing dynamics in the nearshore environment—have been less studied. Here we present results from a high-frequency (once per 30 minutes) FIB sampling event conducted within a Central California, USA, harbor over 48 hours. FIB concentrations at this enclosed site were more variable at high-frequencies than what has been reported at open beach sites. Correlation and regression analyses showed FIB concentrations were most strongly associated with chlorophyll *a* concentration, turbidity, wind speed, and tide level. Results indicate the importance of measuring FIB concentrations and explanatory environmental parameters at appropriate temporal resolutions when conducting water quality monitoring or source tracking studies. Overall, this work highlights how high-frequency sampling can effectively provide information about water quality dynamics at beaches of interest.

## Introduction

Beaches are monitored worldwide for fecal indicator bacteria (FIB) in order to assess microbial water quality. FIB have been linked to the presence of enteric pathogens and exposure to elevated levels of FIB may correspond to increased risk of illnesses such as gastroenteritis and respiratory infection [1, 2]. It is estimated that up to 90 million illnesses and costs of over $2 billion annually can be attributed to poor water quality at United States (US) beaches [3]. Thus, it is important to conduct routine monitoring in order to assess immediate health risk and inform the public, and guide remediation efforts of chronically contaminated sites [4].

In the US, most municipal health agencies sample beaches in their jurisdictions approximately once per week or less often [5, 6]. However, sampling at this temporal resolution may not be sufficient to understand beach water quality dynamics. FIB fate and transport are governed by environmental drivers like precipitation, solar irradiation, tidal forcing, and currents (among others), all of which vary on finer temporal scales [7, 8]. Single, infrequently-collected samples may not represent current FIB levels (or those hours later), leading to inaccurate water quality assessment and public notification [9]. However, it is untenable to continuously monitor FIB concentrations. While physical sensors can provide instantaneous measurements of parameters like temperature, salinity, or pressure (and thus can log data at a frequency < 1 Hz if needed), there are no such sensors for FIB. FIB concentrations are typically measured using culture-based methods that require up to 24 hours to complete. While rapid test methods such as qPCR-based assays are increasingly utilized to measure FIB [10], those assays still require approximately 6 hours for completion on top of the time needed for sample collection, sample transportation, and result dissemination. Moreover, beach management agencies have limited fiscal and staffing resources which prevent higher-frequency monitoring of beaches [5].

Given these constraints, strategic sampling based on knowledge of FIB variability in the environment and its drivers is needed. Because environmental parameters like tide, solar irradiance, winds, and water temperature are either easily measurable or predictable, discerning their role in FIB fate and transport at a given beach can help in designing proactive sampling programs and in developing predictive models which can provide more frequent water quality data [11, 12]. Yet for the many US beaches that are sampled weekly or less frequently, many years would be needed to build a dataset that contains the range of each environmental parameter sufficient to do so. Alternatively, high-frequency sampling over short durations of time could be an effective methodology for acquiring the data needed to understand FIB variability at a beach. This method involves sampling water at regular, short intervals (e.g., once every 30 min) for 1–2 days to collect FIB and environmental data across diverse meteorological and oceanic conditions. There have been some studies that use this technique to study variability of FIB concentrations in beach water [13–15]. Boehm (2007) measured *Enterococcus* concentrations in marine water at ten minute intervals at two California beaches, identifying ‘patchy’ behavior in the FIB concentrations and significant, yet site-varying, associations between FIB concentrations and tide level [15]. Searcy and Boehm (2021) found that FIB concentrations obtained during a single high-frequency sampling event were sufficient to develop machine learning-based predictive FIB models that perform on par to those developed using years-long datasets [16].

While these studies have provided insight into the temporal variability of FIB at marine beaches, they detail observations collected at ‘open’ beaches—or beaches unimpeded from mixing with offshore waters. Conversely, we are not aware of work examining subhourly FIB variation at ‘enclosed’ beaches such as those within harbors, marinas, estuaries, and lagoons. Enclosed beaches that are barriered from waves and associated currents offer protection for both wildlife to have sanctuary and humans for commercial, cultural, and recreational purposes. However, they can also be subject to chronic FIB contamination [6] likely due to relatively long flushing times [17] and their proximity to human development. Thus there is a need to study the temporal dynamics of water quality at enclosed beaches in order to provide guidance on when they should be monitored and how they should be managed differently than open beaches.

The aims of our study were to examine subhourly FIB variability and to determine the extent in which FIB concentrations covary with environmental parameters at an enclosed beach. Here we present the results from a high-frequency sampling event conducted over 48 hours at a Central California harbor beach. We analyze the overall and high-frequency variability in FIB data collected at subhourly intervals during the sampling event. Using correlation and regression analyses, we compare these datasets to simultaneously collected environmental data. Results provide insight into FIB fate and transport at enclosed beaches and can be used to guide future monitoring, modeling, and restoration efforts at these sites.

## Materials and methods

### Study site

**Fig 1:**
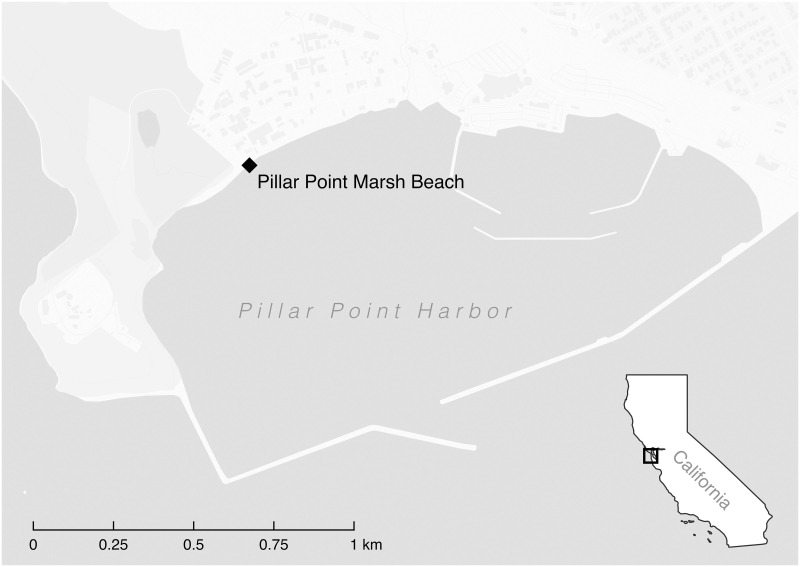
Map of study site at the Pillar Point Harbor. Basemap reprinted under a CC BY license, with permission from CARTO (https://carto.com/), original copyright 2018.

The study was conducted at Pillar Point Marsh Beach (37.502 N, 122.493 W), an approximately 1 km long sandy beach enclosed within the Pillar Point Harbor in Half Moon Bay, California (Fig 1). The beach is one of several recreational beaches contained by the harbor jetties where the public fishes, kayaks, and swims. In recent years, the beaches within Pillar Point Harbor have experienced relatively poor water quality as FIB concentrations frequently rise above the State of California’s regulatory thresholds [6]. As such, the harbor has been the site of a source tracking study [18] and has been declared impaired under the US Clean Water Act [19].

The harbor also supports a sport and commercial fishing fleet, a yacht club, and waterfront restaurants. The land cover surrounding the harbor is a mixture of residential, industrial, agricultural, and undeveloped salt marsh; three creeks drain into the harbor. The region has a Mediterranean climate, with cool, dry summers often with windy or foggy conditions, and slightly colder and relatively wetter winters. Annual precipitation averages approximately 670 mm [20], falling mostly in the months between October and April.

### High-frequency sampling event

We conducted a high-frequency sampling event at the beach site. Between 8:00a PST August 1^st^, 2022 to 7:30a PST August 3^rd^, 2022, water samples were collected every 30 minutes (N = 96 samples). An additional sequence of samples was collected between 11:01a PST and 11:29 PST on August 2^nd^, 2022 (chosen due to available sampling personnel) at a rate of 1 per minute (N = 29 samples); this sequence of sampling will be referred to hereafter as the ‘sprint’ sampling event (as opposed to the ‘main’ sampling event).

Immediately upon collection, samples were stored in the dark and on ice. Samples were processed in batches every six hours using IDEXX defined substrate assays (IDEXX, Westbrook, ME) to enumerate three FIB: total coliforms (TC), *Eschericia coli* (EC), and *Enterococcus* (ENT). TC and EC were measured using Colilert-18, and ENT using Enterolert. 10 ml of environmental sample water was diluted into 90 ml of pH 7.2 Butterfield’s phosphate buffer (Thermo Scientific, Waltham, MA) along with the IDEXX reagent, deposited into IDEXX Quanti-Tray 2000s, and incubated at the vendor-specified temperatures for the required length of time. After incubation, samples were read according to the manufacturer’s instructions and recorded in units of Most Probable Units (MPN)/100 mL. Using this method, the lower and upper limit of detection (LOD) of all three FIB were 10 and 24,196 MPN/100 mL, respectively. A negative control blank consisting of 90 mL of phosphate buffer and 10 mL of distilled water was prepared during each batch; all controls measured below the LOD for FIB.

Additional water quality parameters were collected at each 30-minute sampling point. Water temperature, salinity, and chlorophyll *a* concentration were measured using a YSI 6600 multiparameter water quality sonde (YSI Incorporated, Yellow Springs, OH). Turbidity was measured using a portable turbidimeter (HF Scientific, Fort Myers, FL) and solar irradiance was measured using a spectroradiometer (International Light Technologies, Peabody, MA). Calibration procedures for each instrument are described in the Supporting Information.

We aggregated third-party tide and meteorological data collected from internet sources to supplement the field data. Tide level data were provided by NOAA (https://tidesandcurrents.noaa.gov/) and were collected in six-minute intervals at the San Francisco station (approx. 35 km from the site). Meteorological data were available in 30-minute intervals and were provided by the National Centers for Environmental Information (https://www.ncei.noaa.gov/) and collected at the Half Moon Bay airport (1 km from the site); parameters included air and dew point temperature, wind speed and direction, air pressure, precipitation, visibility, and cloud coverage category. Third-party data sources provided quality assurance information on their websites to verify data veracity; specific station details are provided in S1 Table. No data describing the discharge of the creeks into the harbor was available.

There was no need to obtain permission for conducting this study (including field sampling and lab processing) because water sample collection at our study site is not prohibited, and no animals were captured or killed in our experiment. All raw data collected and collated as part of this study are available at the Stanford Digital Repository (https://purl.stanford.edu/ns310jc1934).

### Data processing and analysis

The sampling event data were digitized by manually entering values into spreadsheets in duplicate by a single technician for quality assurance purposes. When the duplicate data entries did not agree, the discrepancy was resolved by a second technician by referencing the original data sheets from the laboratory. FIB data were then merged with the third-party data to form a complete dataset. All subsequent data processing and analyses were performed using the Python programming language (Python 3.7.6, python.org).

FIB concentrations measured below the lower LOD (i.e. < 10 MPN/100 mL) were replaced with a value of 1 MPN/100 mL to better facilitate identification and analysis; no samples measured above the upper LOD. It is to be noted that this choice of replacement value does not affect the hurdle modeling conducted below. FIB concentrations were categorized as to whether they exceeded the State of California’s regulatory standards for marine waters [21]. For TC and ENT, the exceedance thresholds are 10,000 MPN/100 mL and 104 MPN/100 mL, respectively; there is no such limit for *E*. *coli*, so we used the regulatory limit for fecal coliform (400 MPN/100 mL) to categorize EC samples because California agencies use both *E*. *coli* and fecal coliform interchangeably for beach management [12]. FIB concentrations were log_10_ transformed prior to statistical inference and modeling to reduce skewness and variability.

No missing values were found in the water temperature, tide, visibility, and cloud coverage data. Less than 5% (N = 4) of the values of each of the remaining environmental parameters were identified missing. Missing data points were replaced with values linearly interpolated from neighboring values in the time series. Chlorophyll *a* concentration and turbidity were log_10_-transformed to reduce skewness and variability; because these parameters can be zero-valued, a value of 1 was added to each data point prior to log10-transformation. Solar irradiance data were not collected between sunset and sunrise; data points during these times were assigned a value of 0 W/m^2^. Tide level was classified as high or low if it was above or below 1 meter above mean lower low water, respectively. Alongshore and offshore wind speeds were calculated from raw wind speed and direction values and the angle normal to the beach (140 degrees from true north). Cloud coverage was converted into dummy variables representing three categories: clear, overcast, and partly cloudy.

Measures of central tendency and dispersion (e.g., means, medians, and standard deviations) were calculated. Variation in the FIB data was assessed in multiple ways. We calculated the coefficient of variation (CV, defined as the ratio between the standard deviation and mean) to assess variability across the entire sampling event. High-frequency variability was determined by calculating the normalized difference in concentration between adjacent samples (defined as δ_i_(t) = |c(t+i)–c(t)| / μ, where i is the sampling interval (e.g. 30 minutes), c(t) and c(t+i) are the FIB concentrations of samples collected at time t and t + i, and μ is the mean FIB concentration across the entire time series. In addition to calculating δ_i_ for both the 30-minute interval ‘main’ sampling event data (δ_30m_) and the ‘sprint’ data (δ_1m_), we also calculated δ_i_ using downsampled versions of the 30-minute interval dataset to identify how variability of FIB changes with different resolution sampling rates. This involved resampling the data at intervals of 1, 2, 3, 6, and 12 hours. Because resampling the overall dataset to lower frequencies yields multiple choices of where the resampled dataset can start, there were multiple resampled datasets available from which to calculate δ_i_. For example, resampling the data to an interval of 1 hour yielded two datasets: one where the first sample occurred at 8:00a and one where the first sample occurred at 8:30a. Thus, we calculated the mean and standard deviation of δ_i_ across all resampled datasets for each interval.

While δ_i_ was used to assess the concentration difference in adjacent samples, we also determined the proportion of samples in which the detection status (i.e., FIB measured above or below the LOD) differed between two adjacent samples; the same was done for the regulatory exceedance status (i.e. FIB measured above or below the CA State threshold) of adjacent samples. Finally, we assessed the extent of serial correlation in the FIB concentrations using the partial autocorrelation function. While we attempted to apply other common time series techniques such as autoregressive-moving average (ARMA) models and spectral analysis, no illuminating patterns emerged and thus the analyses were omitted from the manuscript for brevity.

Associations between the FIB data and environmental parameters were assessed; the following analyses were conducted using the 30-minute interval FIB data only. Correlations were calculated using Spearman’s rank correlation. To determine temporal lag effects on FIB, cross-correlation analysis was conducted between FIB and environmental observations temporally shifted up to 3 hours. We categorized FIB concentrations by temporal environmental conditions including if the sample was collected during the day or night, during high or low tide level (i.e. greater or less than 1m relative to mean lower low water), and cloud category. Comparisons of central tendency between environmental categorizations of FIB concentrations were performed using the Kruskal−Wallis test.

Descriptive models (intended to capture associations instead of determine causality [22]) were developed using the FIB and environmental variable datasets. The aforementioned environmental parameters collected during the sampling event and from third-party sources were used as model inputs (i.e. independent variables). In addition to their values observed at the time of FIB sample collection, we also used temporally-lagged versions of these environmental variables with a lag of up to three hours prior to represent potentially delayed effects of an environmental driver on FIB concentrations. To reduce multicollinearity in the modeling dataset, we kept only the version of each environmental variable with the largest magnitude Spearman rank correlation with FIB concentrations. For example, because dew point temperature measured 30 minutes prior to sampling had the highest Spearman rank correlation to ENT concentrations, the remaining lagged versions of the dew point temperature variable were excluded. Finally, the number of hours away from solar noon and the time of day (i.e. daytime or nighttime) were also included in the modeling dataset. Variables were normalized prior to use in modeling by first subtracting their mean value and dividing by their standard deviation; thus the variable coefficients in the following models provide a relative measure of their importance to explaining FIB behavior.

Because samples measured below the LOD were present in the FIB data (i.e. the datasets were zero-inflated), we used a ‘hurdle’ model to model FIB concentrations. Hurdle models treat the probability of occurrence of an event and the magnitude of an event separately [23, 24]. As such, hurdle models are ‘two-part models’ where the first part classifies a binary outcome of whether an event has occurred and the second part estimates the magnitude of the event given that it has occurred. In this case, the first (binary) model classified whether a FIB sample was measured above the LOD, and the second (regression) model estimated the FIB concentration in a sample given that it was measured above the LOD.

We used a random forest classifier as the binary model to predict whether FIB in a sample was above the LOD. Random forests (RF) are ensemble models where the prediction is an aggregate of the predictions of multiple decision ‘trees’; we chose to use RFs because they are non-parametric (i.e. they make no underlying assumptions about the distributions of the data [25] and they yield additional information describing the relative importance of model ‘features’ (i.e. variables) [26]. RFs were fit using the RandomForestClassifer algorithm with the *scikit-learn* package in Python. The default parameters of the algorithm were used, including 100 trees per forest, bootstrap resampling when fitting individual trees, and a random subset of variables per tree split equivalent to the square root of the total number of features [27]. In order to reduce feature importance bias [28], the final set of variables were selected by assessing each variable’s importance using a *permutation feature importance* algorithm [27, 29]. The permutation feature importance of a given variable is indicative of how dependent a model is on that variable and is calculated as the change in model accuracy upon fitting a model after randomly shuffling the variable’s data five times. Variables with a mean permutation feature importance equal to 0 were removed and the final RF was fit using the remaining. The resulting feature importance values were thus normalized to the final variable set.

In the second part of the hurdle models, FIB concentration (given that it is above the LOD) was modeled with generalized least squares (GLS) regression. GLS models are an extension of ordinary least squares models but are equipped to address serial correlation in the model residuals. We implemented the *GLSAR* algorithm with the *statsmodels* package in Python, and used a constant intercept, five iterations to achieve model convergence, and a second-order autoregressive structure to model the residuals as we found significant serial correlation to second order in the FIB data. The output of the GLS models was log_10_-transformed FIB concentration. Models were initially fit using all independent variables and variance inflation factors (VIFs) were calculated to ensure the assumption of variable independence in linear regression was met. If variables had VIFs than 5, the variable with the greatest VIF was removed and the model was refit. This process was repeated until the VIF of each variable was less than 5. Final model coefficients and their p-values were further assessed to determine the effect of environmental variables on FIB concentrations; coefficients with p-values less than 0.05 were considered significantly different than 0. To further determine if assumptions of linear regression were met, the Durbin-Watson statistic was assessed to gage the independence of regression model residuals.

Each FIB was modeled separately; thus, three total models were developed. For EC and ENT, model predictions were made as the product of the binary part and the regression part of the hurdle model. Because no TC samples were measured below the LOD, only the regression part of the hurdle model was applied to model TC concentrations.

## Results

### High-frequency sampling event data

**Fig 2:**
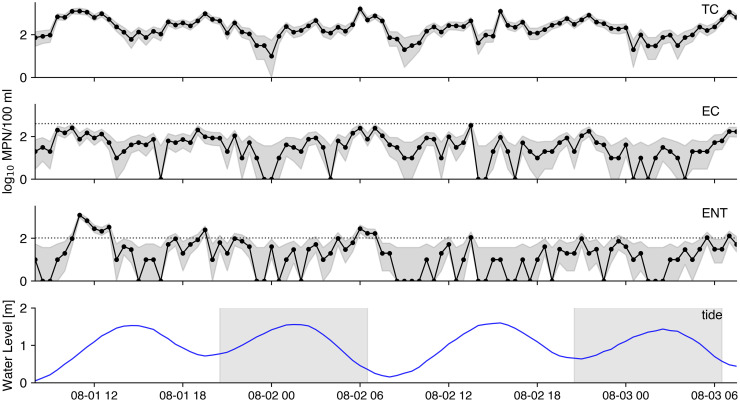
Time series of FIB data collected during the sampling event. Log_10_ transformed TC, EC, and ENT concentrations are presented in the top three subplots. Gray area surrounding the points represents the 95% confidence interval. The dashed lines represent the regulatory threshold, and samples below the LOD are plotted with a value of 0. Tide level during each sampling point is plotted in the final subplot where gray shading represents periods during the night.

We collected 125 samples during the high-frequency sampling event. Over 48 hours, we measured FIB in 96 samples collected during the ‘main’ sampling event at a rate of 1 per 30 minutes (Table 1 and Fig 2). Mean TC, EC, and ENT concentrations during the main sampling event were 319, 60, and 62 most probable number (MPN)/100 ml, respectively. TC, EC, and ENT concentrations were below the LOD in 0, 10, and 24 samples, respectively. ENT exceeded the CA regulatory threshold in 12 samples; TC and EC did not exceed their regulatory thresholds. Serial correlation in the FIB data was insignificant after one hour lag (S1 Fig). Strong bivariate correlation (Spearman ⍴ > 0.5) in concentration was found between all three FIB types (S2 Table).

**Table 1:** Summary of FIB data collected every 30 minutes during the ‘main’ sampling event.

FIB	TC	EC	ENT
**N**	96	96	96
**EXC**	0	0	12
**Below LOD**	0	10	24
**mean**	319	60	62
**max**	1607	327	1212
**std**	328	65	150
**CV**	1.03	1.08	2.4
**δ**	0.6 (0–4.1)	0.8 (0–5.4)	1.0 (0–17.8)

N—number of samples, EXC—number of FIB regulatory standard exceedances, mean/max—mean and maximum FIB concentration (in MPN/100 mL) std—standard deviation (in MPN/100 mL), CV—coefficient of variation, δ—normalized difference between adjacent samples collected 30 minutes apart (i.e. δ_30m_). Here, the mean δ_30m_ is presented along with the range in parentheses.

An additional 29 samples were collected at a rate of 1 per minute during the ‘sprint’ sampling event between (Table 2 and S2 Fig). Including the samples collected at the top and bottom of the hour (i.e. at 1100 and 1130), mean TC, EC, and ENT concentrations in the ‘sprint’ sampling event data were 1251, 191, and 20 MPN/100ml, respectively. In 12 of 29 (39%) samples ENT concentrations were below the LOD; EC and TC concentrations were all above the LOD. No sprint sample exceeded the FIB regulatory thresholds.

**Table 2:** Summary of FIB data collected every 1 minute during the ‘sprint’ sampling event.

FIB	TC	EC	ENT
**N**	31	31	31
**EXC**	0	0	0
**Below LOD**	0	0	12
**mean**	1251	191	20
**max**	3255	389	97
**std**	906	105	23
**CV**	0.72	0.55	1.17
**δ**	0.4 (0–1.6)	0.4 (0–1.1)	0.8 (0–3.8)

N—number of samples, EXC—number of FIB regulatory standard exceedances, mean/max—mean and maximum FIB concentration (in MPN/100 mL) std—standard deviation (in MPN/100 mL), CV—coefficient of variation, δ—normalized difference between adjacent samples collected 1 minute apart (i.e. δ_1m_). Here, the mean δ_1min_ is presented along with the range in parentheses.

Mean water and air temperatures during the sampling event were 15.1°C and 15.3°C, respectively. Mean salinity was 34.0 and ranged from 33.5 to 34.6. Mean chlorophyll *a* concentration was 20.8 μg/L and ranged from 0.1 to 178.6 μg/L. Mean turbidity was 58.1 nephelometric turbidity units (NTU) and ranged from 3.4 to 256.0 NTU. The minimum and maximum tide level was 0.1 and 1.6 m relative to mean lower low water. The mean and maximum wind speed was 3.2 and 7.2 m/s. Cloud category was overcast for 43% of the sampling event, clear for 43%, and partly cloudy for the remainder. Time series of the environmental parameters measured during the sampling events are plotted in S3 Fig.

### Variation in FIB concentrations

ENT concentrations were variable than TC and EC concentrations (Tables 1 and 2). Considering the ‘main’ sampling event data (i.e. samples collected every 30 minutes) (Table 1), the CV for ENT concentrations (2.40) was more than twice that for TC (1.03) and EC (1.08). Variation between adjacent samples was high; mean δ_30m_ for ENT concentrations was 1.0 and ranged from 0 to 17.8. In other words, ENT concentrations between consecutive samples varied, on average, the equivalent of the mean concentration across the entire experiment and nearly twenty-fold that in one incident. There were 30 samples in which the ENT quantification status (i.e whether the sample was above or below the LOD) changed in the following sample; there were 12 samples in which the ENT exceedance status changed. Finally, there were 15 samples in which ENT concentrations did not change in adjacent samples.

There was less variability in FIB concentrations during the 1-minute interval sprint sampling event compared to during the main sampling event data based on CV and δ (Table 2). For example, the CV of ENT measured during the sprint sampling event was 1.17 while the mean δ_1m_ was 0.8 (and ranged from 0–3.8). There were 7 (23%) ENT samples in which the detection status changed in the following sample while no ENT samples changed in exceedance status. Finally, there were 12 samples (39% of samples) in which ENT did not change concentration in adjacent samples in the sprint data.

**Fig 3:**
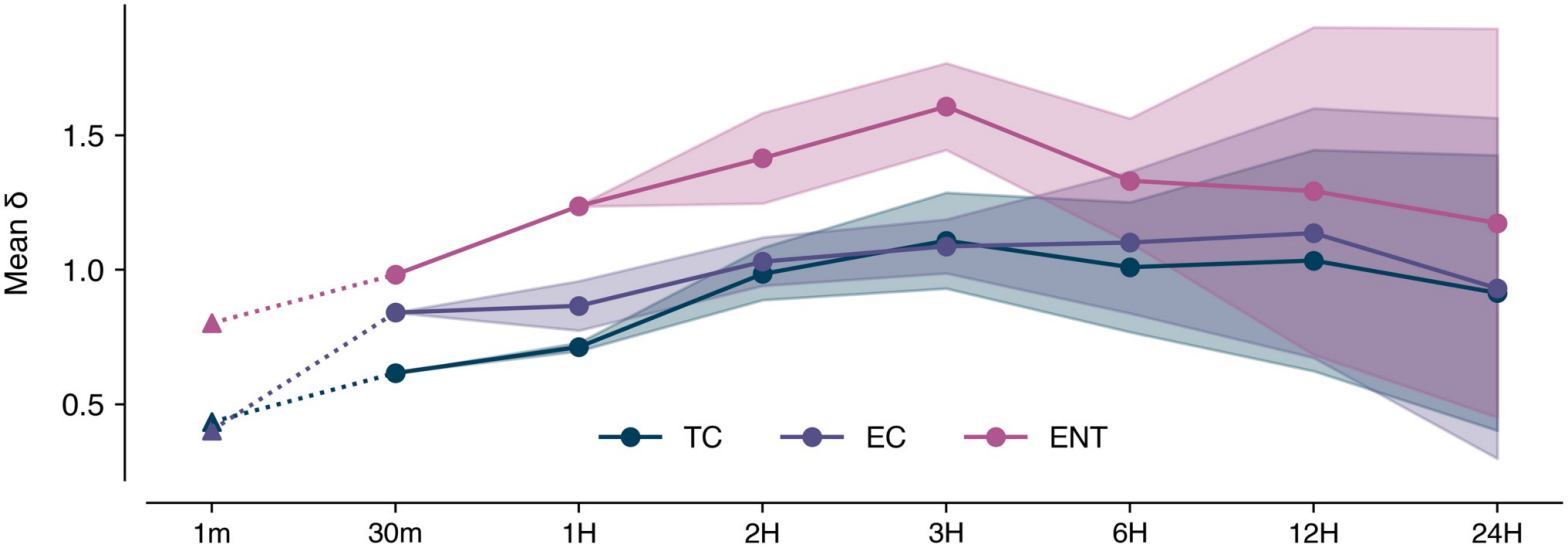
Downsampling analysis. Mean δ_i_ was calculated using the sprint data (i = 1m) as well as samples downsampled from a 30 minute sampling interval (30m) to one hour (1H), two hour (2H), three hour (3H), six hour (6H), 12 hour (12H), and 24 hour (24H) intervals. As downsampling interval increases, there are multiple choices of the first sampling time point. As such, the mean value across all versions of the downsampled data for a given interval (dots) as well as the standard deviation of those values (shaded regions) are plotted.

Assessment of δ_i_ calculated using a range of sampling intervals (i.e. the 1 per minute sprint dataset, the 30-minute interval main sampling event dataset, and the downsampled datasets) showed that the amount of change between adjacent FIB samples increased as sampling interval increased (Fig 3). In other words, samples collected farther apart in time from each other are more different.

### Relationships between FIB and environmental variables

Correlation analysis highlighted the monotonic association between FIB and environmental variables (S3 Table). Concentrations of all three FIB were most strongly associated (|⍴| ≥ 0.2) with turbidity, tide level, and chlorophyll *a* concentration. Cross-correlation analysis indicated that the correlation between FIB and the majority of environmental parameters varied as the time between observations of each increased (S4A–S4C Fig). In some cases (e.g. ENT concentration and tide, chlorophyll *a* concentration, and turbidity), the correlation between FIB and an environmental variable decreased and even switched directions as the time lag between them increased. In other cases (e.g. ENT concentrations and wind speed), the correlation between FIB and environmental variables increased with increasing time lag.

Differences in FIB concentrations were also assessed by grouping samples based on the environmental condition during time of collection. Concentrations of all three FIB were significantly higher during periods of low tide (Kruskal-Wallis p < 0.05). No significant difference was found in FIB concentration measured during the day versus the night, nor between cloud categories (i.e. clear, overcast, or partly cloudy).

We modeled the relationship between FIB concentrations and environmental variables (Tables 3–6). Overall (hurdle) model R^2^ values were 0.46 for TC, 0.82 for EC, and 0.87 for ENT.

**Table 3:** TC descriptive model. The first two columns list each environmental variable and the associated lag with the strongest Spearman correlation to log10-transformed EC concentration. daytime and hours_from_noon were excluded from the temporal lag process. chl and turb values were log10-transformed and all variables were normalized (i.e. made unitless) prior to model fitting. The Concentration Model columns show the variable coefficients and associated p-values of the generalized least squares regression model; values where p-value is less than 0.05 are bolded and variables with no value were removed from the model due to high variance inflation factor (VIF). Note than because TC was never measured below the LOD, no Binary Model (i.e. random forest) was fit.

Model–TC		Concentration Model	
variable	lag (min)	coefficient	p-value
*intercept*	-	**2.249**	**< 0.001**
wtemp	0	0.027	0.734
sal	120	-0.028	0.669
chl*	180	-0.040	0.377
turb*	0	**0.354**	**<0.001**
rad	180	0.039	0.484
tide	30	-	-
tide_high	60	-0.022	0.769
temp	180	-	-
dtemp	0	-0.129	0.128
pres	150	0.026	0.769
wspd	0	-	-
awind	30	-0.045	0.316
owind	180	-0.019	0.799
vis	180	0.029	0.638
cloud: overcast	90	-0.145	0.088
cloud: partly	90	-0.051	0.288
cloud: clear	180	0.028	0.720
daytime	-	-0.153	0.077
hours_from_noon	-	**-**	**-**

**Table 4:** EC descriptive (hurdle) model. The first two columns list each environmental variable and the associated lag with the strongest Spearman correlation to log10-transformed EC concentration. daytime and hours_from_noon were excluded from the temporal lag process. chl and turb values were log10-transformed and all variables were normalized (i.e. made unitless) prior to model fitting. The Binary Model column shows the non-zero permutation feature importances (i.e. the change in model accuracy upon fitting a model after randomly shuffling the variable’s data five times) of the random forest model. The Concentration Model columns show the variable coefficients and associated p-values of the generalized least squares regression model; values where p-value is less than 0.05 are bolded and variables with no value were removed from the model due to high variance inflation factor (VIF).

Hurdle Model–EC		Binary Model	Concentration Model	
variable	lag (min)	importance	coefficient	p-value
*intercept*	-	-	**1.580**	**< 0.001**
wtemp	120	-	**0.234**	**0.001**
sal	120	-	0.086	0.131
chl*	0	-	0.065	0.223
turb*	0	0.038	**0.255**	**0.002**
rad	0	-	-	-
tide	30	0.029	-	-
tide_high	60	-	-0.013	0.847
temp	0	-	-	-
dtemp	0	-	**-0.220**	**0.008**
pres	180	-	-0.113	0.111
wspd	0	-	-	-
awind	30	0.020	-0.031	0.522
owind	0	0.011	-	-
vis	30	-	**0.127**	**0.027**
cloud: overcast	0	-	0.034	0.691
cloud: partly	180	-	-0.072	0.148
cloud: clear	0	-	-0.074	0.388
daytime	-	-	-0.053	0.478
hours_from_noon	-	-	**-**	**-**

**Table 5:** ENT descriptive (hurdle) model. The first two columns list each environmental variable and the associated lag with the strongest Spearman correlation to log10-transformed EC concentration. daytime and hours_from_noon were excluded from the temporal lag process. chl and turb values were log10-transformed and all variables were normalized (i.e. made unitless) prior to model fitting. The Binary Model column shows the non-zero permutation feature importances (i.e. the change in model accuracy upon fitting a model after randomly shuffling the variable’s data five times) of the random forest model. The Concentration Model columns show the variable coefficients and associated p-values of the generalized least squares regression model; values where p-value is less than 0.05 are bolded and variables with no value were removed from the model due to high variance inflation factor (VIF).

Hurdle Model–ENT		Binary Model	Concentration Model	
variable	lag (min)	importance	coefficient	p-value
*intercept*	-	-	**1.494**	**< 0.001**
wtemp	30	0.027	-0.010	0.887
sal	180	-	-0.002	0.980
chl*	0	0.044	**0.200**	**0.002**
turb*	0	0.036	0.074	0.436
rad	60	-	-0.108	0.104
tide	60	-	-	-
tide_high	60	-	-0.095	0.181
temp	90	-	-	-
dtemp	90	-	-	-
pres	120	-	0.104	0.183
wspd	0	-	-	-
awind	150	0.022	-0.109	0.102
owind	30	0.002	0.080	0.399
vis	180	0.002	-0.028	0.630
cloud: overcast	150	-	-	-
cloud: partly	30	-	-0.119	0.146
cloud: clear	180	-	**0.226**	**0.014**
daytime	-	-	-	-
hours_from_noon	-	0.004	**-0.232**	**0.003**

**Table 6:** Descriptive model metrics. The Binary Model columns display the number of samples (out of 96) that were measured below the LOD and the accuracy of the random forest in classifying samples above or below the LOD. Note than because TC was never measured below the LOD, no Binary Model was fit. The Concentration Model columns display the R^2^, root mean square error (RMSE) and Durbin-Watson statistic of the GLS models which predict log10-transformed FIB concentration of samples measured above the LOD. The Overall Model columns display the R^2^ and root mean square error (RMSE) of the hurdle models which predict log10-transformed FIB concentration of all samples.

FIB	Binary Model		Concentration Model			Overall (Hurdle) Model	
	# Below LOD	Accuracy	R^2^	RMSE	Durbin-Watson	R^2^	RMSE
TC	-	-	0.464	0.336	2.032	0.464	0.336
EC	10	1.0	0.373	0.303	2.113	0.819	0.271
ENT	22	1.0	0.419	0.333	2.294	0.866	0.294

Turbidity, tide level, and alongshore and offshore wind speed were important (i.e. had nonzero permutation feature importances) in the EC RF model, while turbidity, chlorophyll *a concentration*, water temperature, visibility, the number of hours from noon, and alongshore and offshore wind speed were important in the ENT RF model. Turbidity was the only significant (i.e. p-value less than 0.05) variable in the TC GLS model. Turbidity, water temperature, dew point temperature, and visibility were significant variables in the EC GLS model while chlorophyll *a* concentration, the number of hours from noon, and whether the sky was clear of clouds were significant variables in the ENT GLS model. Salinity, solar irradiance, air temperature, air pressure, and whether it was day or nighttime were neither important nor significant variables in the hurdle models. Variance inflation factors for all variables were less than 5 and Durbin-Watson statistics were all approximately 2, suggesting that multicollinearity and serial correlation respectively were adequately accounted for.

## Discussion

The goal of this research is to study subhourly FIB variability and to determine the extent in which FIB concentrations covary with environmental parameters at an enclosed marine beach. We conducted a 48-hour sampling event at a Central California harbor beach, analyzing 125 water samples for FIB concentrations and collecting associated tide, water quality, and meteorological data. To our knowledge, this dataset is one of the few multi-day, subhourly FIB datasets that exist and the first of its kind to be collected at an enclosed location.

A main result from our analysis is that the variability of the FIB concentrations collected at enclosed beaches can be greater than at open beaches. For example, the CV and mean δ_30m_ of ENT concentrations collected by Boehm (2007) at Huntington Beach, California (after adjustment from a 10- to 30-min sampling interval) were 1.51 and 0.7, respectively, compared to 2.40 and 1.0 for ENT in this study (S4 Table). Additionally, Searcy and Boehm (2021) reported the CV and mean δ_30m_ of FIB concentrations measured at Cowell Beach, California in 2011 were 1.19 and 0.5 for ENT and 0.92 and 0.4 for EC (compared to 1.08 and 0.8 in this study) (S4 Table). These results confirm that FIB concentrations at enclosed beaches can be highly variable and inconsistent; however, the mechanisms by which this occurs remain to be determined. Variability in FIB concentrations in marine waters can be attributed to intermittent inputs of FIB to the system, dispersion and advection that serve to mix FIB in three dimensions, and variable fate process that might remove the FIB from the system. Intermittent FIB sources and variable fate processes are likely to be present at both open and enclosed marine beaches, however, mixing is likely to be very different at the two types of beaches. A wave-driven surf zone at an open ocean beach causes intense mixing in all three dimensions in the surf zone [30, 31]; a surf zone is absent at an enclosed beach. Its absence could allow gradients in FIB concentrations to persist over the time scales of our sampling. Additional work on circulation would complement studies like this in the future.

High CV and δ values reinforce the notion that management decisions about beach swimming advisories should not be solely based on single, infrequently-collected samples [15]. Due to this variation found in the data, it is unlikely that a single sample represents the mean value across a longer time span (e.g. over the course of a week which is the typical sampling frequency of agencies). Because FIB could be below the LOD in one sample and above the state regulatory threshold 30 minutes later, public notification of risk based on a single sample is not appropriate. Instead, a statistical aggregation of the results of multiple samples collected over different periods in the tidal and solar cycles may be more representative and health protective. Such an aggregation could be a moving average (which would serve to smooth highly variable concentrations that a single sample provides) or the maximum of a group of samples (which would be more health conservative). Though likely unfeasible in the present, managers could begin to manage beaches based on the results of multiple, high-frequency samples as automation of FIB sampling becomes increasingly accessible [32]. Alternatively, high-frequency sampling events can be a relatively efficient method of developing predictive FIB models [16] that rely on more easily attainable environmental data as inputs and can be more accurate than using single samples alone to manage beaches [33].

The difference in variability and concentrations relative to the regulatory thresholds between each of the three FIB measured at Pillar Point Harbor Beach provides additional insights for beach managers. ENT was found to be more variable and in higher relative concentrations than TC and EC, indicating the importance of measuring more than a single indicator of water quality at beaches. Different FIB types can indicate the presence of contamination from different sources, and this may not be captured by monitoring only a single indicator. Further, this measured difference should also reiterate that FIB are not a fully representative indicator of health risk; that is, they do not provide information about water quality that constituents such as heavy metals, pesticides, and microplastics do.

Clear statistical associations between the high-frequency FIB and environmental data help build an understanding of what environmental mechanisms may be important in driving FIB fate and transport at this beach. They may also be useful for determining strategic time periods in which to conduct routine FIB monitoring. For example, the tidal association we observed indicated that higher FIB concentrations may be measured during periods of low tide. Tidal level may influence both the FIB source at this beach (which may be beach groundwater, bird feces on the beach, or runoff from outlets around the harbor) and circulation of water which transports FIB. Wind speed was additionally important, perhaps suggesting that FIB within the harbor is transported by currents driven independently from tides. Surprisingly, solar irradiance–a documented driver of FIB fate [34]—did not appear to be an influential variable, while visibility, hours since noon, and cloud coverage occurred in the descriptive models with mixed effects on FIB concentrations. This may be due to the general lack of clear, sunny skies throughout the experiment or FIB being more strongly driven by other fate and transport mechanisms (e.g. tide and wind driven transportation or when a FIB source exceeds a sink).

Chlorophyll *a* concentration and turbidity were the most strongly correlated environmental parameters to FIB concentrations, and were selected as covariates with positive directionality in all descriptive models. This finding is consistent with other studies of FIB at beaches [33], and could be linked to sediment deposition and resuspension in the swash zone [35, 36]. However, chlorophyll concentration and turbidity are not regularly monitored at California beaches. As they can be more easily measured than FIB using optical sensors, we recommend that beach monitoring programs begin measuring these parameters as they may be useful in conserving sampling resources and increasing the accuracy of predictive FIB models.

Additional work may further highlight the utility of a high-frequency sampling approach. The results presented within come from a single enclosed beach site; additional work is needed at additional enclosed sites to determine the generalizability of these results. While it is the summer, dry season in which beach recreation in California is at its highest, beaches are still utilized by surfers, divers, and fishers in the wet season. A future study could explore the high-frequency variability of FIB before, during, and after the ‘first flush’, or the first major storm event of a year. Runoff due to precipitation is a major source of FIB in beach water [37], so it is imperative that FIB data are collected during wet weather events. Finally, while we have presented initial statistical analysis on the reported data, they could also be used for predictive modeling efforts, to design future source tracking studies, or to inform adaptive sampling programs at the beach whereby FIB is sampled at strategic time points and auxiliary environmental data are collected.

Altogether we found that conducting high-frequency FIB sampling events is an effective mechanism by which to understand beach water quality dynamics. If resources are available to do so, we encourage local governmental agencies, academic groups, tribes, or community groups at beaches worldwide to use a high-frequency sampling framework to rapidly build an initial understanding of FIB variability and its link to environmental drivers. The data collected from such sampling efforts could then be used by communities to develop sampling strategies to manage health risk or inform remediation efforts to abate contamination found at beaches.

## Supporting information

S1 Appendix Instrument calibration procedures. (DOCX)

S1 Fig Partial autocorrelation plots of the FIB time series. The subsequent vertical lines in each subplot indicate the partial autocorrelation coefficient of the log10-transformed FIB time series (y-axes) at increasing time lags (x-axes). Correlations above the grey dashed lines indicate significance as determined by Bartlett’s formula. Calculated using the 30-minute interval ‘main’ campaign data (N = 96 samples).
(DOCX)

S2 Fig Time series of FIB data collected during the ‘sprint’ sampling campaign. Data collected on 2 August 2022 between 1100 and 1130 (N = 31 samples). Log-10 transformed TC, EC, and ENT concentrations are presented in the top three subplots. Gray area surrounding the points represent the 95% confidence interval. The dashed lines represent the regulatory threshold, and samples below the LOD are plotted with a value of 0.
(DOCX)

S3 Fig Environmental parameter time series. wtemp—water temperature; sal—salinity; chl—chlorophyll concentration; turb—turbidity; rad—solar irradiance; tide—tide level; temp—air temperature; wspd—wind speed.
(DOCX)

S4 Fig A. Cross-correlation between TC and environmental parameters. Spearman rank correlations are plotted, and values significant to p < 0.05 are marked with ‘*’. Color indicates the strength and direction of the correlation. tide—tide level; wtemp—water temperature; sal—salinity; turb—turbidity; chl—chlorophyll concentration; rad—solar irradiance; temp—air temperature; dtemp—dew point temperature; pres—air pressure; wspd—wind speed; owind—offshore wind speed; awind—alongshore wind speed. B. Cross-correlation between EC and environmental parameters. Spearman rank correlations are plotted, and values significant to p < 0.05 are marked with ‘*’. Color indicates the strength and direction of the correlation. tide—tide level; wtemp—water temperature; sal—salinity; turb—turbidity; chl—chlorophyll concentration; rad—solar irradiance; temp—air temperature; dtemp—dew point temperature; pres—air pressure; wspd—wind speed; owind—offshore wind speed; awind—alongshore wind speed. C. Cross-correlation between TC and environmental parameters. Spearman rank correlations are plotted, and values significant to p < 0.05 are marked with ‘*’. Color indicates the strength and direction of the correlation. tide—tide level; wtemp—water temperature; sal—salinity; turb—turbidity; chl—chlorophyll concentration; rad—solar irradiance; temp—air temperature; dtemp—dew point temperature; pres—air pressure; wspd—wind speed; owind—offshore wind speed; awind—alongshore wind speed.
(DOCX)

S1 Table A: Environmental data stations for third-party sources. B: Environmental variable considered in multivariate analyses. turb and chl were log_10_-tranformed prior to model fitting. All variables except for daytime and hours_from_noon were temporally lagged up to 3 hours (180 minutes) and correlated to FIB concentrations prior to model fitting.
(DOCX)

S2 Table Spearman rank correlations between FIB types. Calculated using the 30-minute interval ‘main’ campaign data (N = 96 samples).
(DOCX)

S3 Table Spearman rank correlations between FIB and environmental variables. Calculated using the 30-minute interval ‘main’ campaign data (N = 96 samples).
(DOCX)

S4 Table Comparison of variability to reported studies. CV is unitless; δ has units of 1 / 30 minutes.
(DOCX)
