# Supplementary material for: High-frequency fecal indicator bacteria (FIB) observations to assess water quality drivers at an enclosed beach
Source: PLoS One. 2023 Jun 2;18(6):e0286029. doi: 10.1371/journal.pone.0286029 (PMC10237476; doi:10.1371/journal.pone.0286029)
Supplement: S1 Appendix — (DOCX) [file pone.0286029.s001.docx]

**S1 Appendix**

**Instrument Calibration Procedures**

Sensor calibration of the YSI 6600 Multiparameter Water Quality Sonde was completed before the sampling campaign following the calibration guides published in the YSI 6-Series User Manual. A two-point calibration was performed on the conductivity sensor using a 100 mS/cm calibration solution (YSI 3165) with a diluted 10 mS/cm point and a non-diluted 100 mS/cm point. A two-point calibration was performed on the chlorophyll optical sensor using deionized water and a diluted fluorescent dye solution (Kingscote Chemicals, Miamisburg, OH). The Portable Turbidimeter was calibrated before each six-hour batch processing event during the sampling campaign by inserting a Primetime 0.02 NTU Calibration Standard (HF Scientific 60002) into the optical well and manually setting the Reference Adjustment value to 0.02. The Spectroradiometer was calibrated before each reading by performing a dark scan within the associated SpectrILight III Software.
