# Supplementary material for: High-frequency fecal indicator bacteria (FIB) observations to assess water quality drivers at an enclosed beach
Source: PLoS One. 2023 Jun 2;18(6):e0286029. doi: 10.1371/journal.pone.0286029 (PMC10237476; doi:10.1371/journal.pone.0286029)
Supplement: S1 Fig — The subsequent vertical lines in each subplot indicate the partial autocorrelation coefficient of the log10-transformed FIB time series (y-axes) at increasing time lags (x-axes). Correlations above the grey dashed lines indicate significance as determined by Bartlett’s formula. Calculated using the 30-minute interval ‘main’ campaign data (N = 96 samples). (DOCX) [file pone.0286029.s002.docx]

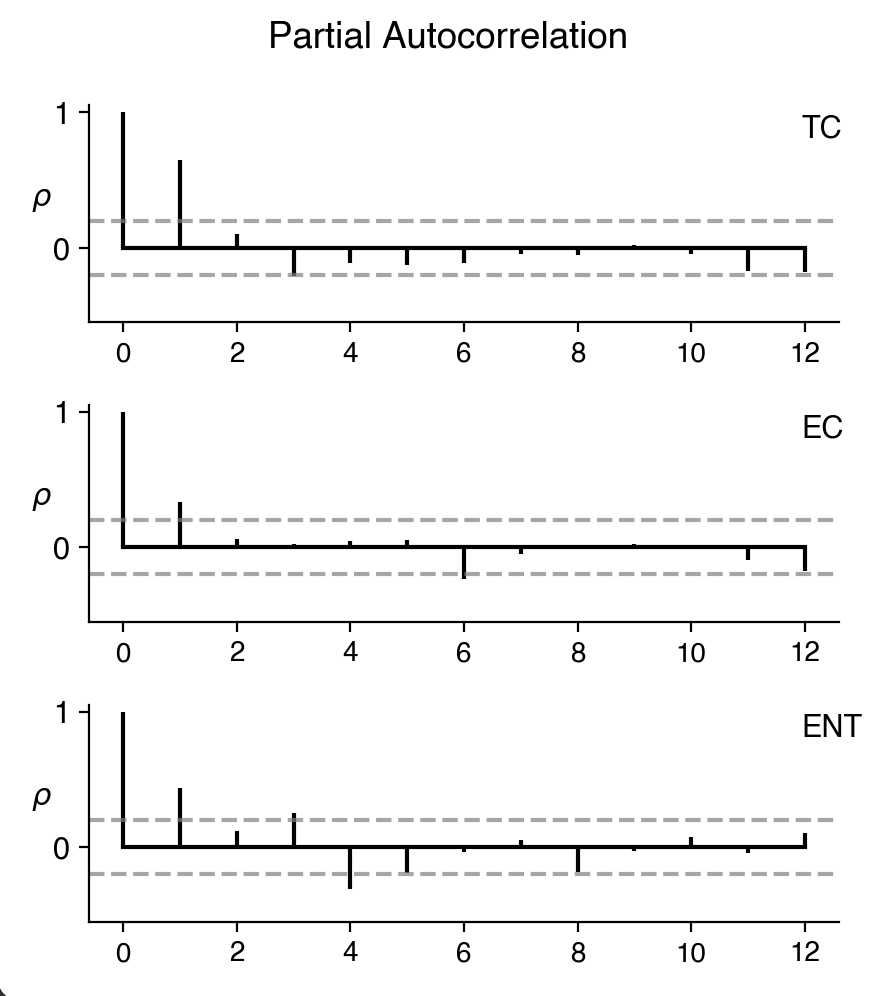


S1 Figure: Partial autocorrelation plots of the FIB time series. The subsequent vertical lines in each subplot indicate the partial autocorrelation coefficient of the log10-transformed FIB time series (y-axes) at increasing time lags (x-axes). Correlations above the grey dashed lines indicate significance as determined by Bartlett’s formula. Calculated using the 30-minute interval ‘main’ campaign data (N=96 samples).
