# Supplementary material for: High-frequency fecal indicator bacteria (FIB) observations to assess water quality drivers at an enclosed beach
Source: PLoS One. 2023 Jun 2;18(6):e0286029. doi: 10.1371/journal.pone.0286029 (PMC10237476; doi:10.1371/journal.pone.0286029)
Supplement: S2 Fig — Data collected on 2 August 2022 between 1100 and 1130 (N = 31 samples). Log-10 transformed TC, EC, and ENT concentrations are presented in the top three subplots. Gray area surrounding the points represent the 95% confidence interval. The dashed lines represent the regulatory threshold, and samples below the LOD are plotted with a value of 0. (DOCX) [file pone.0286029.s003.docx]

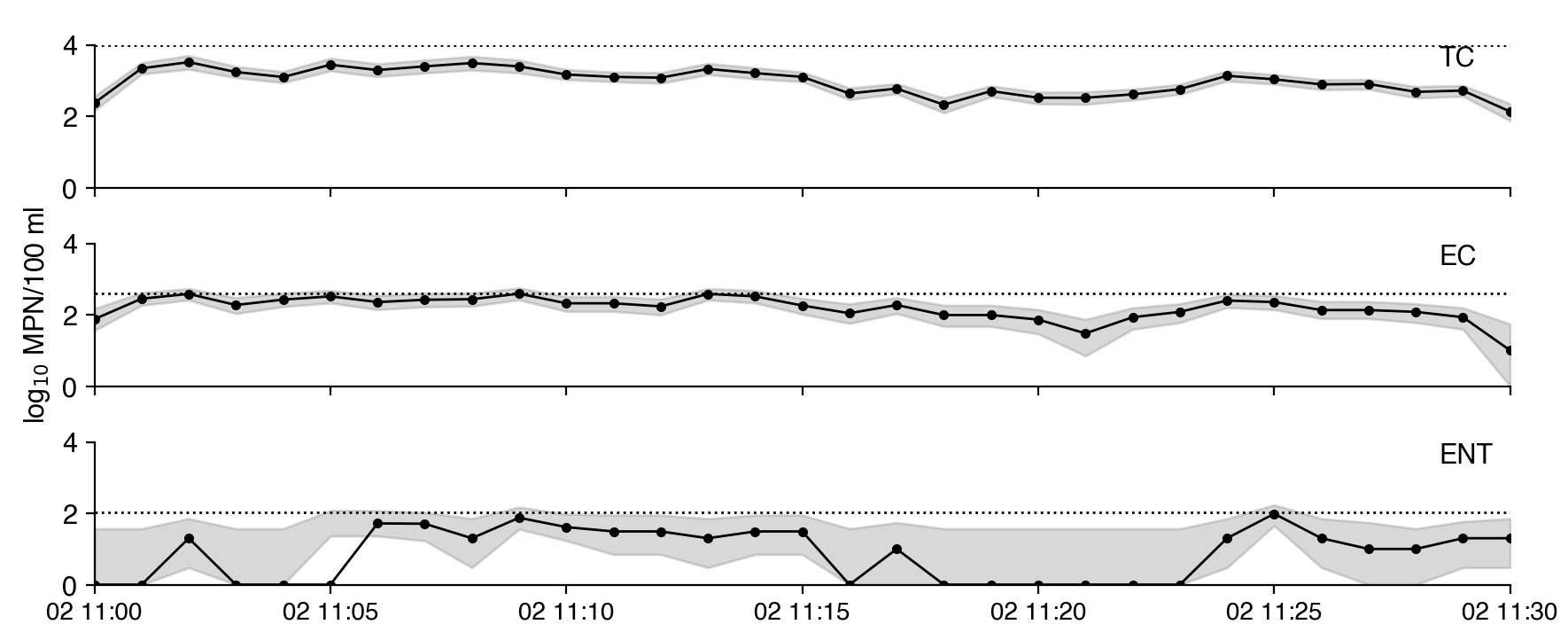


S2 Figure. Time series of FIB data collected during the ‘sprint’ sampling campaign. Data collected on 2 August 2022 between 1100 and 1130 (N=31 samples). Log-10 transformed TC, EC, and ENT concentrations are presented in the top three subplots. Gray area surrounding the points represent the 95% confidence interval. The dashed lines represent the regulatory threshold, and samples below the LOD are plotted with a value of 0.
