# Supplementary material for: High-frequency fecal indicator bacteria (FIB) observations to assess water quality drivers at an enclosed beach
Source: PLoS One. 2023 Jun 2;18(6):e0286029. doi: 10.1371/journal.pone.0286029 (PMC10237476; doi:10.1371/journal.pone.0286029)
Supplement: S3 Fig — wtemp—water temperature; sal—salinity; chl—chlorophyll concentration; turb—turbidity; rad—solar irradiance; tide—tide level; temp—air temperature; wspd—wind speed. (DOCX) [file pone.0286029.s004.docx]

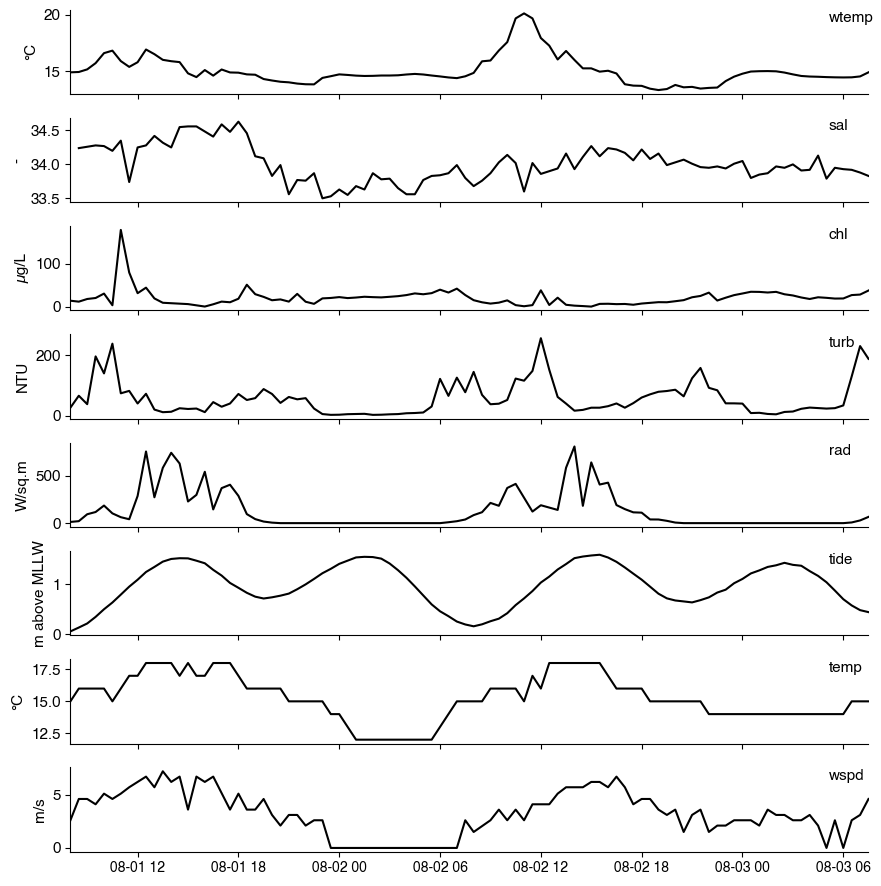


S3 Figure. Environmental parameter time series. wtemp - water temperature; sal - salinity; chl - chlorophyll concentration; turb - turbidity; rad - solar irradiance; tide - tide level; temp - air temperature; wspd - wind speed.
