# Supplementary material for: High-frequency fecal indicator bacteria (FIB) observations to assess water quality drivers at an enclosed beach
Source: PLoS One. 2023 Jun 2;18(6):e0286029. doi: 10.1371/journal.pone.0286029 (PMC10237476; doi:10.1371/journal.pone.0286029)
Supplement: S4 Fig — A. Cross-correlation between TC and environmental parameters. Spearman rank correlations are plotted, and values significant to p < 0.05 are marked with ‘*’. Color indicates the strength and direction of the correlation. tide—tide level; wtemp—water temperature; sal—salinity; turb—turbidity; chl—chlorophyll concentration; rad—solar irradiance; temp—air temperature; dtemp—dew point temperature; pres—air pressure; wspd—wind speed; owind—offshore wind speed; awind—alongshore wind speed. B. Cross-correlation between EC and environmental parameters. Spearman rank correlations are plotted, and values significant to p < 0.05 are marked with ‘*’. Color indicates the strength and direction of the correlation. tide—tide level; wtemp—water temperature; sal—salinity; turb—turbidity; chl—chlorophyll concentration; rad—solar irradiance; temp—air temperature; dtemp—dew point temperature; pres—air pressure; wspd—wind speed; owind—offshore wind speed; awind—alongshore wind speed. C. Cross-correlation between TC and environmental parameters. Spearman rank correlations are plotted, and values significant to p < 0.05 are marked with ‘*’. Color indicates the strength and direction of the correlation. tide—tide level; wtemp—water temperature; sal—salinity; turb—turbidity; chl—chlorophyll concentration; rad—solar irradiance; temp—air temperature; dtemp—dew point temperature; pres—air pressure; wspd—wind speed; owind—offshore wind speed; awind—alongshore wind speed. (DOCX) [file pone.0286029.s005.docx]

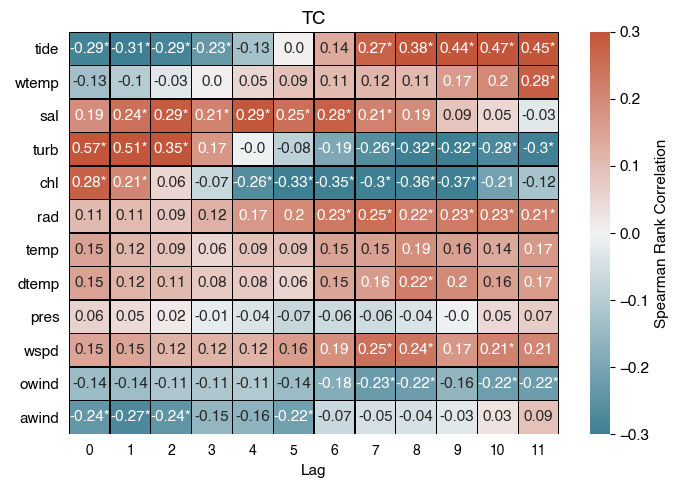


S4 Figure A. Cross-correlation between TC and environmental parameters. Spearman rank correlations are plotted, and values significant to p < 0.05 are marked with ‘*’. Color indicates the strength and direction of the correlation. tide - tide level; wtemp - water temperature; sal - salinity; turb - turbidity; chl - chlorophyll concentration; rad - solar irradiance; temp - air temperature; dtemp - dew point temperature; pres - air pressure; wspd - wind speed; owind - offshore wind speed; awind - alongshore wind speed.


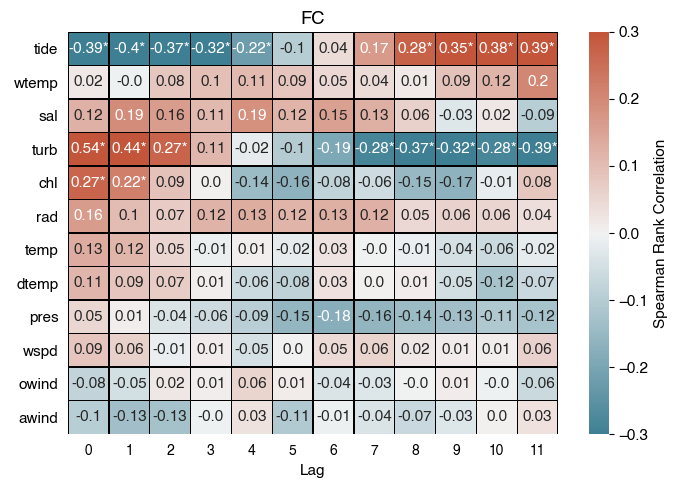


S4 Figure B. Cross-correlation between EC and environmental parameters. Spearman rank correlations are plotted, and values significant to p < 0.05 are marked with ‘*’. Color indicates the strength and direction of the correlation. tide - tide level; wtemp - water temperature; sal - salinity; turb - turbidity; chl - chlorophyll concentration; rad - solar irradiance; temp - air temperature; dtemp - dew point temperature; pres - air pressure; wspd - wind speed; owind - offshore wind speed; awind - alongshore wind speed.


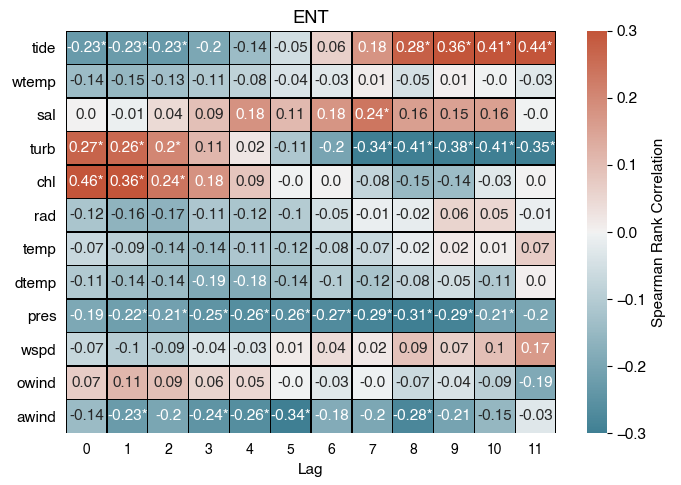


S4 Figure C: Cross-correlation between TC and environmental parameters. Spearman rank correlations are plotted, and values significant to p < 0.05 are marked with ‘*’. Color indicates the strength and direction of the correlation. tide - tide level; wtemp - water temperature; sal - salinity; turb - turbidity; chl - chlorophyll concentration; rad - solar irradiance; temp - air temperature; dtemp - dew point temperature; pres - air pressure; wspd - wind speed; owind - offshore wind speed; awind - alongshore wind speed.
