# Supplementary material for: High-frequency fecal indicator bacteria (FIB) observations to assess water quality drivers at an enclosed beach
Source: PLoS One. 2023 Jun 2;18(6):e0286029. doi: 10.1371/journal.pone.0286029 (PMC10237476; doi:10.1371/journal.pone.0286029)

S1 Table A: Environmental data stations for third-party sources


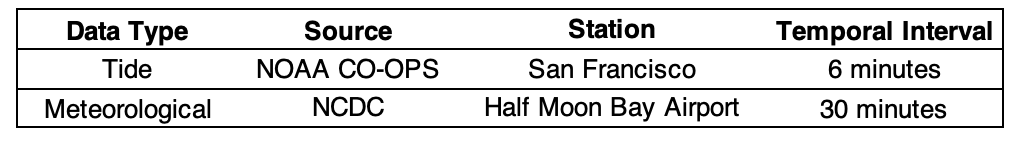


S1 Table B: Environmental variable considered in multivariate analyses. turb and chl were log_10_-tranformed prior to model fitting. All variables except for daytime and hours_from_noon were temporally lagged up to 3 hours (180 minutes) and correlated to FIB concentrations prior to model fitting.


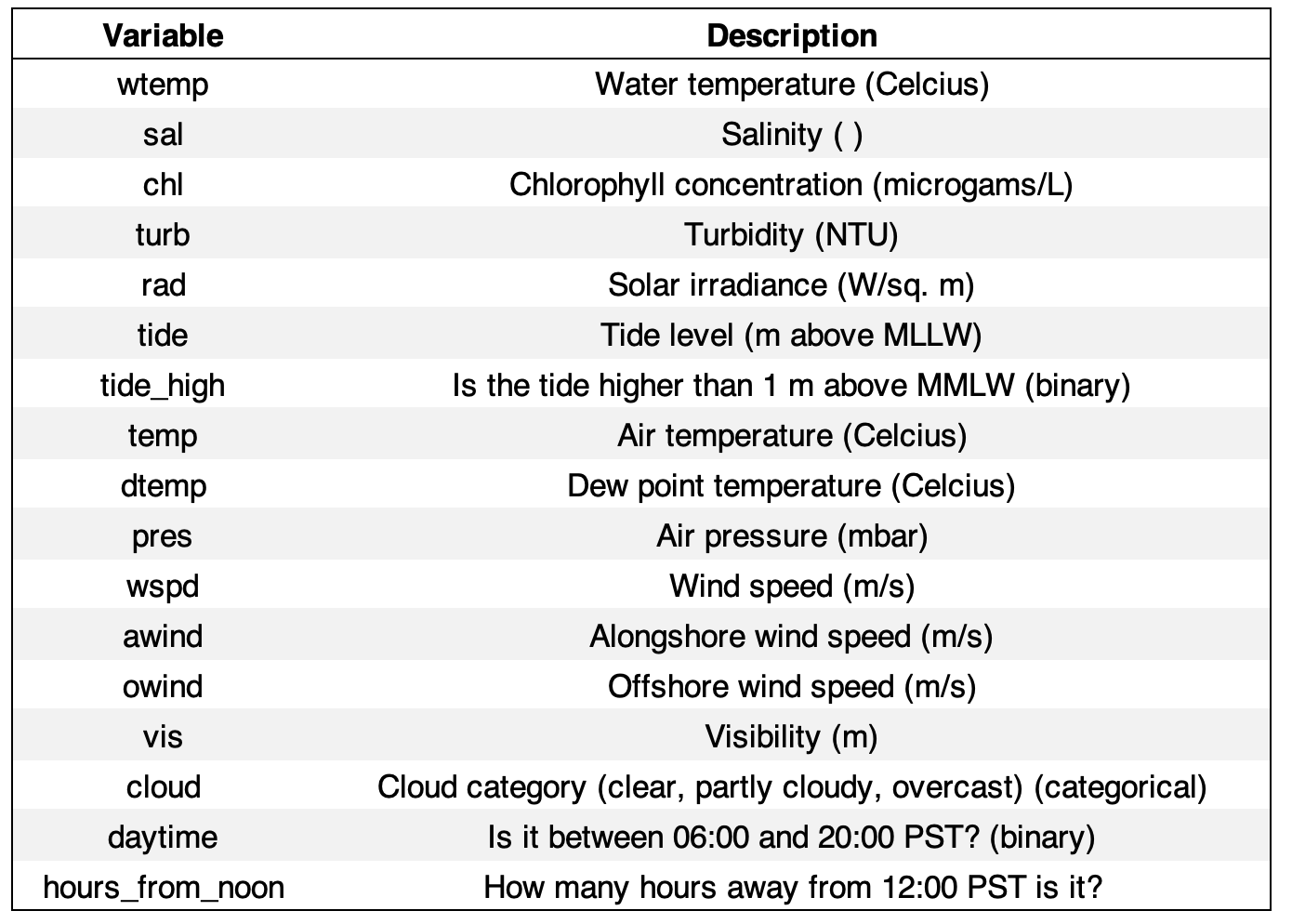

Supplement: S1 Table — A: Environmental data stations for third-party sources. B: Environmental variable considered in multivariate analyses. turb and chl were log10-tranformed prior to model fitting. All variables except for daytime and hours_from_noon were temporally lagged up to 3 hours (180 minutes) and correlated to FIB concentrations prior to model fitting. (DOCX) [file pone.0286029.s006.docx]
