# Supplementary material for: High-frequency fecal indicator bacteria (FIB) observations to assess water quality drivers at an enclosed beach
Source: PLoS One. 2023 Jun 2;18(6):e0286029. doi: 10.1371/journal.pone.0286029 (PMC10237476; doi:10.1371/journal.pone.0286029)

S3 Table: Spearman rank correlations between FIB and environmental variables. Calculated using the 30-minute interval ‘main’ campaign data (N=96 samples).


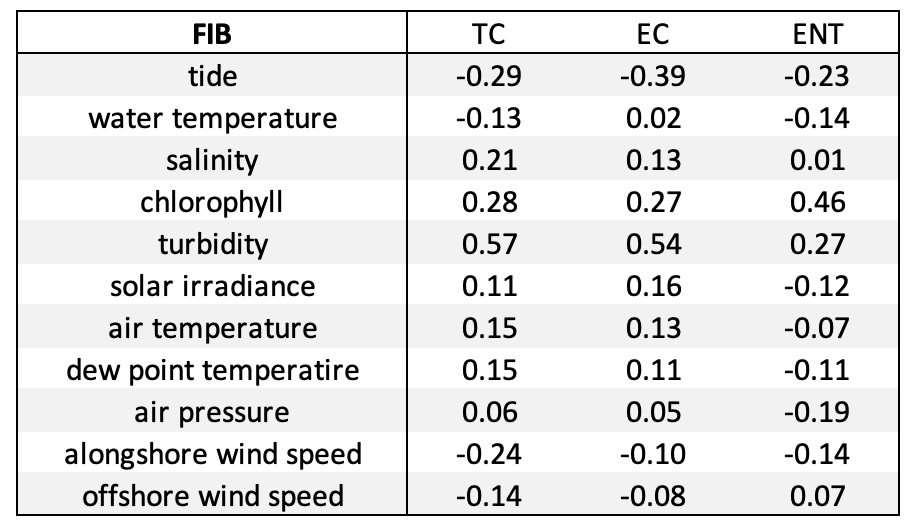

Supplement: S3 Table — Calculated using the 30-minute interval ‘main’ campaign data (N = 96 samples). (DOCX) [file pone.0286029.s008.docx]
