# Supplementary figures and images for: High-frequency fecal indicator bacteria (FIB) observations to assess water quality drivers at an enclosed beach
Source: PLoS One. 2023 Jun 2;18(6):e0286029. doi: 10.1371/journal.pone.0286029 (PMC10237476; doi:10.1371/journal.pone.0286029)

S4 Table. Comparison of variability to reported studies. CV is unitless; δ has units of 1 / 30 minutes.


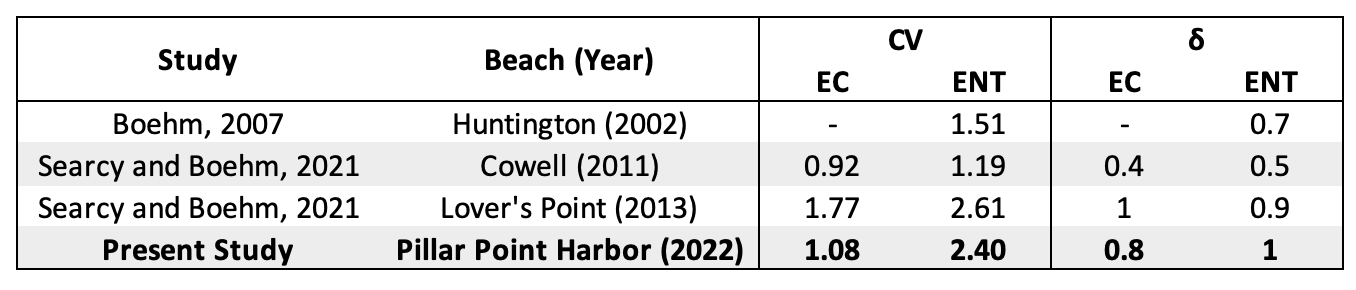

Supplement: S4 Table — CV is unitless; δ has units of 1 / 30 minutes. (DOCX) [file pone.0286029.s009.docx]
